# T4 Phage Displaying Dual Antigen Clusters Against H3N2 Influenza Virus Infection

**DOI:** 10.3390/vaccines13010070

**Published:** 2025-01-13

**Authors:** Shenglong Liu, Mengzhou Lin, Xin Zhou

**Affiliations:** 1 College of Veterinary Medicine, Institute of Comparative Medicine, Yangzhou University, Yangzhou 225009, China; liusl801@163.com (S.L.); linmengzhouabc@163.com (M.L.); 2 Jiangsu Co-Innovation Center for Prevention and Control of Important Animal Infectious Diseases and Zoonoses, Yangzhou 225009, China; 3 Joint International Research Laboratory of Agriculture and Agri-Product Safety, The Ministry of Education of China, Yangzhou 225009, China

**Keywords:** T4 phage nanovaccine, HA1 and M2e antigen molecule clusters, immune response

## Abstract

Background: The current H3N2 influenza subunit vaccine exhibits weak immunogenicity, which limits its effectiveness in preventing and controlling influenza virus infections. Methods: In this study, we aimed to develop a T4 phage-based nanovaccine designed to enhance the immunogenicity of two antigens by displaying the HA1 and M2e antigens of the H3N2 influenza virus on each phage nanoparticle. Specifically, we fused the Soc protein with the HA1 antigen and the Hoc protein with the M2e antigen, assembling them onto a T4 phage that lacks Soc and Hoc proteins (Soc^−^Hoc^−^T4), thereby constructing a nanovaccine that concurrently presents both HA1 and M2e antigens. Results: The analysis of the optical density of the target protein bands indicated that each particle could display approximately 179 HA1 and 68 M2e antigen molecules. Additionally, animal experiments demonstrated that this nanoparticle vaccine displaying dual antigen clusters induced a stronger specific immune response, higher antibody titers, a more balanced Th1/Th2 immune response, and enhanced CD4^+^ and CD8^+^ T cell effects compared to immunization with HA1 and M2e antigen molecules alone. Importantly, mice immunized with the T4 phage displaying dual antigen clusters achieved full protection (100% protection) against the H3N2 influenza virus, highlighting its robust protective efficacy. Conclusions: In summary, our findings indicate that particles based on a T4 phage displaying antigen clusters exhibit ideal immunogenicity and protective effects, providing a promising strategy for the development of subunit vaccines against various viruses beyond influenza.

## 1. Introduction

Influenza, a respiratory disease worthy of attention, annually accounts for approximately one billion cases of seasonal influenza, exerting a significant burden on global public health and socio-economic systems [1]. As the primary culprit behind influenza, the influenza virus has a segmented RNA genome and can infect multiple species, including humans, birds, and pigs [2], and it exhibits a high mutation rate due to the lack of proofreading mechanisms for its negative-sense RNA genome during replication [3]. This frequent variation makes vaccine-based prevention strategies more challenging and presents significant hurdles for vaccine development [4,5]. The H3N2 influenza virus is an important subtype of the influenza A virus, having historically caused multiple pandemics and seasonal influenza outbreaks. Its hemagglutinin and neuraminidase proteins exhibit relatively high mutation rates, allowing the virus to continually alter its antigenic properties and evade recognition and attack by the host immune system [6]. Moreover, studies have shown that the H3N2 influenza virus can generate new variants through genetic reassortment with other influenza virus subtypes [7]. Since the pandemic in 1968, the transmission of H3N2 has continued to persist [8]. Currently, vaccination remains the primary strategy for controlling influenza viruses [5]. Subunit vaccines are known for their high safety profile, the ability to be rapidly produced at scale with batch-to-batch consistency, and their ease of storage and transportation [9,10,11,12,13,14]. However, traditional subunit vaccines often exhibit a limited capacity to elicit potent immune responses, typically falling short in providing adequate protection [11,15,16,17,18,19,20,21]. Nanoparticles have emerged as a promising class of vaccine carriers, enhancing the uptake of nanoparticle-based vaccines by antigen-presenting cells (APCs) through the mimicry of pathogen-associated molecular patterns (PAMPs) [22,23,24].Consequently, the development of novel, faster, and more scalable nanoparticle-based vaccine technologies can significantly benefit rapid responses to the emergence and pandemics of new influenza strains.

As the most abundant organisms on Earth, phages possess the ability to coexist with a variety of other organisms and are considered highly safe because they cannot infect eukaryotic cells [25]. These viral particles are typically very stable under various harsh environmental conditions and can be produced inexpensively and on a large scale using simple bacterial culture media [26]. Based on these characteristics, vaccine design strategies based on phage display technology have attracted widespread attention from researchers in recent years, with various phages already being used as vaccine carriers in the development of novel vaccines [27,28,29,30]. Phage display technology is highly flexible and precise, allowing multiple antigens to be displayed simultaneously on a single phage, thus providing the possibility for the development of multivalent vaccines [31]. Moreover, phages themselves act as natural adjuvants, and the display of antigens on the phage surface can mimic the natural process of viral infection. As a result, these vaccines can induce a stronger immune response in vivo [32,33]. The non-essential Hoc and Soc proteins on the T4 phage head can be expressed in vitro and still bind with high affinity to the Soc^−^Hoc^−^T4 phage particle [33]. Due to this unique property, the T4 phage can be utilized by researchers to achieve high-density, multi-component antigen display [34,35]. Furthermore, antigens displayed on the T4 phage can be genetically engineered to modify the density or combination of antigen presentation to optimize the immune response [29]. Additionally, T4 phages can simultaneously display multiple different antigenic epitopes, thereby inducing a broader and more comprehensive immune protection [36]. Numerous studies have shown that antigens displayed on the T4 phage surface can elicit a strong immune response even without the use of additional adjuvants [37]. This clearly demonstrates the powerful potential of T4 phages in vaccine delivery [38]. This suggests that we can design a nanoparticle vaccine based on the T4 phage that displays multiple protective antigens.

Hemagglutinin (HA), a glycoprotein on the surface of influenza viruses, is a key antigen in the host’s response to both natural infection and vaccination and serves as the primary component in the development of seasonal influenza vaccines [39]. A complete HA consists of a homotrimer linked by a single disulfide bond, with each monomer composed of two subunits (HA1 and HA2) [40]. HA1 contains the receptor-binding site on the cell surface and most of the neutralizing antibody epitopes [41]. Due to the high mutability of the HA head, most current influenza vaccines focused on optimizing neutralizing antibody responses against the major surface antigen hemagglutinin lose their protective effect [42,43]. Therefore, there is a growing consideration of combining HA with other influenza antigens to create more effective vaccines [44]. M2e, the extracellular domain of the matrix protein M2 of the influenza virus, is a small ion channel membrane protein that is highly conserved across different strains of influenza A viruses and is frequently utilized as the antigenic component in the development of universal influenza vaccines [45,46]. Therefore, designing a fusion protein of HA1 and M2e antigen clusters for display on the T4 phage is considered feasible.

**Scheme 1:**
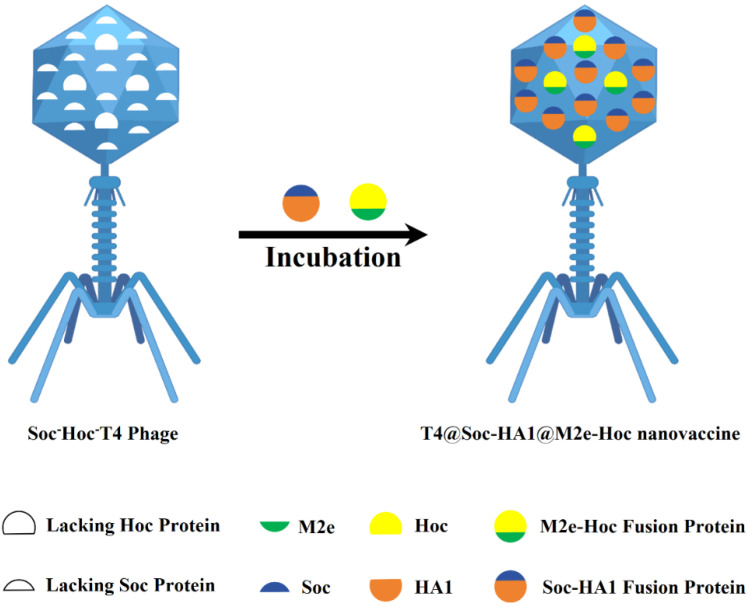
Schematic diagram of T4 phage-based nanovaccine displaying dual antigen clusters. The HA1 and M2e antigens of the H3N2 influenza virus were fused with the Soc and Hoc protein to generate the Soc-HA1 and M2e-Hoc fusion proteins, respectively. These two fusion proteins were co-incubated in vitro with the Soc^−^Hoc^−^T4, resulting in the formation of the T4@Soc-HA1@M2e-Hoc nanovaccine.

Currently, the H3N2 virus continues to be prevalent in animal populations and is also showing a trend of spreading among humans, increasing the likelihood of zoonotic transmission [47]. In this study, we aim to develop a nanoparticle vaccine specifically targeting the H3N2 virus. This vaccine leverages phage display technology to simultaneously present the influenza virus HA1 and M2e antigen clusters on the T4 phage (Scheme 1). This approach not only enables a faster and more efficient production of influenza vaccines but also enhances the immunogenicity of the vaccine, thereby improving protection against the H3N2 virus. Through this innovative vaccine design, we aim to overcome the limitations of traditional influenza vaccines and provide a novel, efficient solution for global influenza prevention and control.

## 2. Experimental Sections

### 2.1. Ethical Statement

All animal procedures in this study were conducted in accordance with the “Regulations for the Administration of Affairs Concerning Experimental Animals” approved by the State Council of the People’s Republic of China. All animal protocols were reviewed and approved by the Animal Welfare and Ethics Committee of Yangzhou University (Approval Number: 202407021).

### 2.2. Experimental Materials Such as Bacterial Strains and Virus Strains

The virus strain used in this research is a strain of A/Hong Kong/4801/2014 (H3N2) preserved in the laboratory; competent cells were all purchased from WEIDI (Shanghai, China); the plasmids pET-28a-M2e-Hoc used as the template for gene amplification, the vector plasmids pET-28a-Soc, pET-28a, and pCold I, as well as the Soc^−^Hoc^−^T4 phage and its host bacteria (*E. coli* BL21), were all preserved in our laboratory; Madin-Darby Canine Kidney (MDCK) cells were purchased from the American Type Culture Collection (ATCC No. CCL-34) and preserved in our laboratory. The experimental animals used were 6–8 week-old female BALB/c mice purchased from the Experimental Animal Center of Yangzhou University in Jiangsu, China.

### 2.3. Construction of Recombinant Expression Vectors

Specific primers were designed to amplify the HA1 and M2e-Hoc (M2e peptides: SLLTEVETPIRNEWGCRCNDSSD) gene fragments via PCR. The amplified fragments were then individually inserted into plasmid vectors to create the expression plasmids pET-28a-Soc-HA, pET-28a-HA, and Pcold-M2e-Hoc. These three expression plasmids were chemically transformed into competent *E. coli* BL21 (DE3) cells. The transformed strains were confirmed by PCR and gene sequencing. Notably, a 6 × His-tag was added at appropriate positions in each of the three target proteins to facilitate subsequent protein purification. Details of all primers can be found in the Supplementary Materials (Table S1).

### 2.4. In Vitro Expression and Purification of Target Proteins

In this study, three target proteins were expressed using an *E. coli* expression system. Initially, the expression strains obtained in Section 2.3 were inoculated at a 1:100 dilution into 2 × YT medium supplemented with antibiotics. The cultures were grown until they reached the logarithmic growth phase, at which point an inducer was added to initiate protein expression. Following a specified induction period, bacterial cells were harvested by centrifugation and washed. The cell pellets were then subjected to ultrasonic disruption to release the expressed proteins. Target proteins were subsequently purified using a His-tag protein purification kit (denaturant-resistant, Beyotime, Shanghai, China), and the purification efficiency was assessed through SDS-PAGE analysis. For proteins expressed in the form of inclusion bodies, renaturation was performed by sequentially employing protein renaturation buffers containing 4 M, 2 M, 1 M, and 0 M urea. The concentrations of HA1, Soc-HA1, and M2e-Hoc proteins were determined using a Bradford protein assay kit (Beyotime, Shanghai, China). Finally, the proteins were aliquoted and stored at −80 °C for future use.

### 2.5. Amplification and Purification of Soc^−^Hoc^−^T4 Phage

Activated *E. coli* BL21 host cells were inoculated into 2 × YT medium at a 1:100 ratio and cultured in a shaker incubator at 37 °C and 220 rpm until the OD reached approximately 0.6. The Soc^−^Hoc^−^T4 phage was then added, and the culture was continued for an additional 6–8 h. The supernatant was collected by centrifugation. The supernatant containing progeny Soc^−^Hoc^−^T4 phage was filtered through a 0.45 μm filter and then subjected to ultracentrifugation at 95,000× *g* for 2 h. The resulting pellet was resuspended in an appropriate amount of PBS buffer and purified by CsCl density gradient ultracentrifugation [48]. The CsCl densities used ranged from low to high: 1.33 g/mL, 1.45 g/mL, 1.5 g/mL, and 1.7 g/mL. The purified Soc^−^Hoc^−^T4 phage was dialyzed to remove residual CsCl, and its titer was determined using the double-layer agar plaque assay [49]. The purified phage was stored at 4 °C for future use.

### 2.6. Validation of In Vitro Assembly of Nanovaccine

The nanovaccines T4@Soc-HA1@M2e-Hoc, T4@Soc-HA1, and T4@M2e-Hoc were obtained by co-incubating fusion proteins with the Soc^−^Hoc^−^T4 phage. Briefly, approximately 5 × 10^10^ Soc^−^Hoc^−^T4 phage particles were added to each 1.5 mL centrifuge tube. An excess of purified M2e-Hoc and Soc-HA1 fusion proteins was added based on the number of Soc and Hoc protein binding sites on the Soc^−^Hoc^−^T4 phage. The reaction volume was adjusted to the same final volume with PBS buffer and incubated at 37 °C for 1 h. After incubation, the mixture was centrifuged at 19,000× *g* for 1 h to remove unassembled fusion proteins. The supernatant and pellet were collected after each centrifugation, and the assembly results were verified using Western blot (WB), targeting the 6×His-tag on the fusion proteins.

During the in vitro assembly validation using ELISA, the Soc^−^Hoc^−^T4 phage was coated onto the ELISA plates overnight as the antigen. After blocking, two types of fusion proteins were added to the ELISA plates in a manner similar to that of antibodies. Subsequently, a mouse-derived 6×His antibody was used as the primary antibody, and an HRP-conjugated goat anti-mouse antibody was used as the secondary antibody. The OD450 was then read using a microplate reader to validate the in vitro assembly results.

### 2.7. Validation of In Vitro Assembly Efficiency

The assembly efficiency was determined using grayscale analysis [32]. Nanovaccines were assembled according to the method described in Section 2.6. The assembled samples were then analyzed by SDS-PAGE. Gradient concentrations of BSA were used as standards. Image J 1.52p software was used to quantify gp23* (49 kDa, with 930 copies per individual phage particle) and gp18 (70 kDa, with 138 copies per individual phage particle) in each lane, thereby calculating the exact number of recombinant phages in the sample. Simultaneously, the same method was used to quantify the M2e-Hoc and Soc-HA1 fusion proteins in the sample. Finally, the in vitro assembly efficiency of the phage was calculated.

### 2.8. Immunization

**Figure 1:**
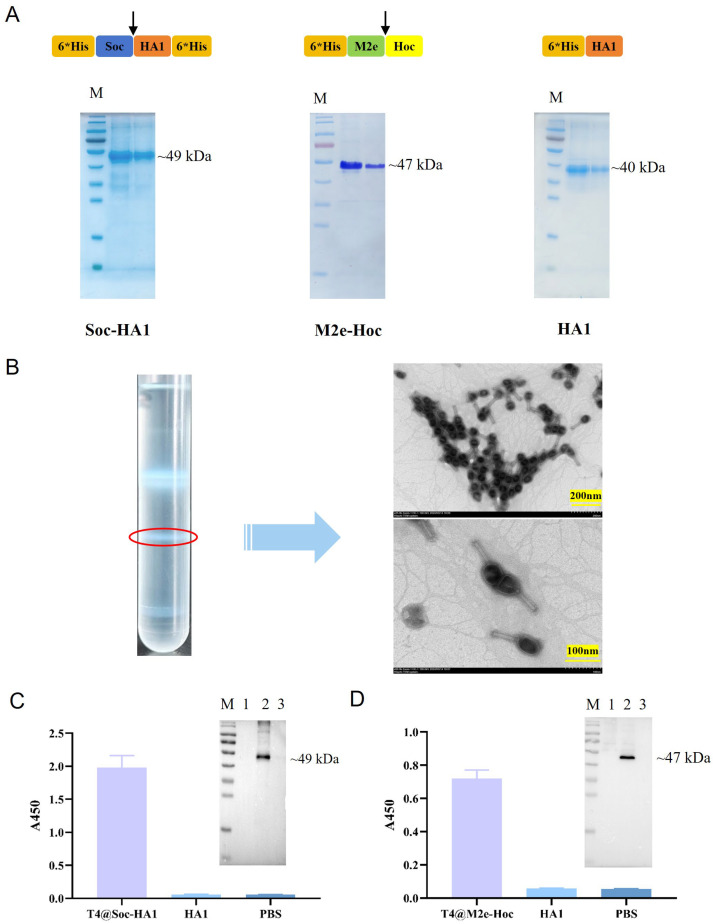
Construction of the nanovaccine. (**A**) Expression and purification of Soc-HA1, M2e-Hoc, and HA1 proteins. The black arrows indicate flexible linkers between the two proteins (M: marker). (**B**) Purification and TEM characterization of Soc^−^Hoc^−^T4 phage. The red circle denotes the purified Soc^−^Hoc^−^T4 phage, while the arrow indicates the characterization of this Soc^−^Hoc^−^T4 phage under TEM. (**C**) Validation of in vitro assembly of T4@Soc-HA1 nanoparticles; confirmed using ELISA and Western blot analysis (Lane M: marker, Lane 1: Soc^−^Hoc^−^T4 phage, Lane 2: T4@Soc-HA1 particles assembled and centrifuged pellet, Lane 3: supernatant after washing and centrifugation). (**D**) Validation of in vitro assembly of T4@M2e-Hoc nanoparticles; confirmed using ELISA and Western blot analysis (Lane M: marker, Lane 1: Soc^−^Hoc^−^T4 phage, Lane 2: T4@M2e-Hoc particles assembled and centrifuged pellet, Lane 3: supernatant after washing and centrifugation).

In this study, we determined the number of mice in each group based on specific requirements and after reviewing multiple related studies [50,51,52]. The three types of nanovaccines prepared according to the method in Section 2.5 (T4@Soc-HA1@M2e-Hoc, T4@Soc-HA1, T4@M2e-Hoc), as well as the soluble M2e peptide, soluble HA1 protein, and PBS buffer, were used to immunize mice via intramuscular injection following the schedule shown in Figure 1A. Specifically, each mouse received distinct immunizations: the nanovaccine group was administered approximately 10^11^ PFU of phage particles, the soluble antigen group received 10 μg of soluble antigen, and the control group was given 100 μL of PBS buffer.

### 2.9. Serum ELISA for Detection of Specific Antibodies

Blood samples were collected in the second week after the third immunization to measure the levels of specific antibodies in the serum using ELISA. In brief, ELISA plates were coated overnight with the HA1 protein and M2e peptide, respectively. After blocking, serially diluted mouse serum was added as the primary antibody, and diluted horseradish peroxidase (HRP)-conjugated goat anti-mouse antibody was used as the secondary antibody. After color development with TMB substrate solution, the OD450 was measured using a microplate reader.

### 2.10. Hemagglutination Inhibition (HAI) Assay

The collected sera from each group were treated overnight with receptor-destroying enzyme (RDE) II and heat-inactivated at 56 °C for 30 min. The inactivated sera were then serially diluted with sterile PBS buffer. Next, the diluted sera were added to a disposable “V”-shaped 96-well plate (25 μL/well), followed by the addition of 4 units of prepared virus (25 μL/well) to each well, and incubated at 37 °C for 30 min. Subsequently, 1% chicken red blood cells (25 μL/well) was added to each well and incubated again at 37 °C for 30 min. Finally, the hemagglutination was observed and recorded [53].

### 2.11. Cytokine Detection

Fourteen days after the third immunization, spleens were harvested from the immunized mice to prepare splenocyte suspensions. These splenocytes were then seeded into 96-well plates at a density of 2 × 10^6^ cells per well, with each sample replicated in four wells. The cells were stimulated with a mixture of HA1 and M2e proteins (10 μg/well). Positive control wells (stimulated with PMA) and negative control wells (stimulated with medium) were also included. Following the addition of the stimulants, the splenocytes were incubated at 37 °C for 18 h. Subsequently, the cells were centrifuged at 500× *g* for 5 min. The levels of IFN-γ and IL-4 cytokines in the supernatants were then measured using a mouse IFN-γ/IL-4 Double Antibody Sandwich ELISA Detection Kit (Proteintech).

Briefly, all necessary reagents and gradient standards were prepared according to the product instructions. For IFN-γ detection, the gradient standards had concentrations of 1000 pg/mL, 500 pg/mL, 250 pg/mL, 125 pg/mL, 62.5 pg/mL, and 31.25 pg/mL. For IL-4 detection, the gradient standards were 250 pg/mL, 125 pg/mL, 62.5 pg/mL, 31.25 pg/mL, 15.6 pg/mL, and 7.8 pg/mL. The required strips of the ELISA plate were taken out per the experiment’s needs, and the assay was performed according to the instructions. Finally, the absorbance at 450 nm was measured using a microplate reader, and the cytokine concentrations were calculated based on standard curves generated from the OD values of the standard wells.

### 2.12. Flow Cytometry

After adjusting the spleen cell counts as described in Section 2.11, the cells were cultured and stimulated using the same method. The stimulated spleen cells were washed with sterile PBS buffer and centrifuged at 500× *g* for 5 min, followed by discarding the supernatant. CD3, CD4, and CD8a monoclonal antibodies (mAbs) were diluted in PBS buffer containing 0.5% BSA at a ratio of 1:200. The stimulated spleen cells from each group were divided into two subgroups, and 50 μL of the diluted antibodies was added to each well. The cells were incubated in the dark at 4 °C for 30 min. After incubation, the cells were centrifuged, the supernatant was discarded, and the cells were washed twice with sterile PBS buffer. Finally, the cells were analyzed using a flow cytometer.

### 2.13. Evaluation of Challenge Protection Efficacy

Two weeks after the third immunization, the mice were challenged intranasally with a dose of 5×LD_50_. The body weight of the mice was recorded daily, and the mortality rate was monitored. Mice with a body weight loss exceeding 30% were defined as dead and euthanized. The observation period continued for 14 days.

### 2.14. Determination of Lung Viral Load and Histopathological Analysis

Mice from each group were euthanized by cervical dislocation five days post-challenge, and their lungs were harvested. The left lung was fixed in 4% paraformaldehyde, then processed through trimming, dehydration, embedding, sectioning, staining, and mounting. The histological sections were observed under a microscope for basic pathological changes. Meanwhile, the right lung was placed in a 1.5 mL centrifuge tube and homogenized at 4 °C with a certain volume of sterile PBS buffer proportional to the lung tissue weight. The lung homogenate was serially diluted 1:10 in a 96-well cell culture plate containing DMEM medium. Subsequently, 100 μL of DMEM containing 2.5 × 10^5^ MDCK cells was added to each well and incubated in a cell culture incubator. After overnight incubation, the DMEM was discarded, and 200 μL of fresh DMEM was added to each well for further incubation for three days. The cytopathic effects and hemagglutination were observed and recorded. The final viral load was calculated using the Reed–Muench method.

### 2.15. Statistical Analysis

Unless otherwise specified, all data are presented as the mean ± standard deviation (SD), and all statistical analyses were performed using GraphPad Prism 8.0 (GraphPad Software). The normality of data distributions for each group was assessed using the D’Agostino–Pearson test and the Shapiro–Wilk test. Differences between groups were compared using one-way ANOVA. In all cases, a *p*-value of <0.05 was considered statistically significant. Unless otherwise stated, Tukey’s multiple comparisons test was used to determine statistical significance, indicated as follows: *, **, ***, ****, and ns denote *p* < 0.05, *p* < 0.01, *p* < 0.001, *p* < 0.0001, and not statistically significant, respectively.

## 3. Results

### 3.1. Acquisition of Soc-HA1, M2e-Hoc, and HA1 Proteins

Through PCR amplification, we acquired the HA1 gene fragment of the influenza virus (generating a band of approximately 1050 bp) and the M2e-Hoc gene fragment fused with the influenza virus M2e sequence (producing a band of approximately 1200 bp) (Figure S1). After inserting the aforementioned gene fragments into the corresponding vector plasmids, we individually transferred the three expression plasmids into *E. coli* BL21 (DE3) competent cells, thereby obtaining three expression strains: pET-28a-Soc-HA, pET-28a-HA, and pCold-M2e-Hoc. Subsequently, since all three target proteins are fused with a 6×His-tag (Figure 1A), upon the completion of protein expression, we purified the expressed target proteins using the His-tag protein purification kit (denaturant-resistant, Beyotime). Additionally, as the Soc-HA1 protein and the HA1 protein are both expressed in the form of inclusion bodies, after the purification process was concluded, we also carried out protein renaturation on these two proteins. Ultimately, we obtained the three target proteins, Soc-HA1, M2e-Hoc, and HA1. After SDS-PAGE analysis, all three proteins were of the expected size and relatively pure (Figure S1). According to determination by the Bradford protein assay kit (Beyotime), the final concentration of the Soc-HA1 protein was 0.25 mg/mL, that of the M2e-Hoc protein was 0.37 mg/mL, and the final concentration of the HA1 protein was 0.23 mg/mL (Figure S2).

### 3.2. Assembly and Characterization of the Nanovaccine

To obtain highly purified phages for assembly, the concentrated Soc^−^Hoc^−^T4 phages were purified via CsCl density gradient centrifugation and characterized by Transmission Electron Microscopy (TEM) (Figure 1B). To verify whether the expressed fusion proteins and the Soc^−^Hoc^−^T4 phages could assemble in vitro, an excess of Soc-HA1 and M2e-Hoc fusion proteins were incubated with approximately 5 × 10^10^ PFU of Soc^−^Hoc^−^T4 phages. After centrifugation, the samples were analyzed by SDS-PAGE and Western blot. The results demonstrated that lanes containing the nanovaccine exhibited bands corresponding to the fusion proteins (Figure 1C,D), whereas lanes containing only Soc^−^Hoc^−^T4 phages and wash supernatants showed no detectable bands (Figure 1C,D). This demonstrated that the Soc-HA1 and M2e-Hoc fusion proteins were effectively displayed on the Soc^−^Hoc^−^T4 phages. Similarly, the ELISA results demonstrated that in ELISA plates coated with Soc^−^Hoc^−^T4 phages, wells incubated with His-tagged Soc-HA1 and M2e-Hoc fusion proteins were positive, whereas control wells incubated with HA1 protein (containing a 6×His-tag) and PBS were negative. This indicates that the Soc-HA1 and M2e-Hoc fusion proteins can be specifically displayed on the Soc^−^Hoc^−^T4 phage (Figure 1C,D).

To further determine the display efficiency of the two fusion proteins on the Soc^−^Hoc^−^T4 phage, the assembled nanovaccine was subjected to SDS-PAGE analysis alongside gradient concentrations of Bovine Serum Albumin (BSA). After Coomassie Brilliant Blue staining, the bands corresponding to the gradient BSA standard protein, gp18, gp23*, M2e-Hoc, and Soc-HA1 proteins were scanned using Image J software to obtain their grayscale values. A standard curve was established based on the grayscale values of the gradient BSA standard protein (Figure S3). By fitting the grayscale values of the gp18, gp23*, M2e-Hoc, and Soc-HA1 bands into the standard curve and converting the values, it was determined that an average of 179 Soc-HA1 fusion proteins and 68 M2e-Hoc fusion proteins could be displayed per Soc^−^Hoc^−^T4 phage.

### 3.3. Nanovaccine Based on T4 Phage Elicited a Strong Humoral Immune Response

**Figure 2:**
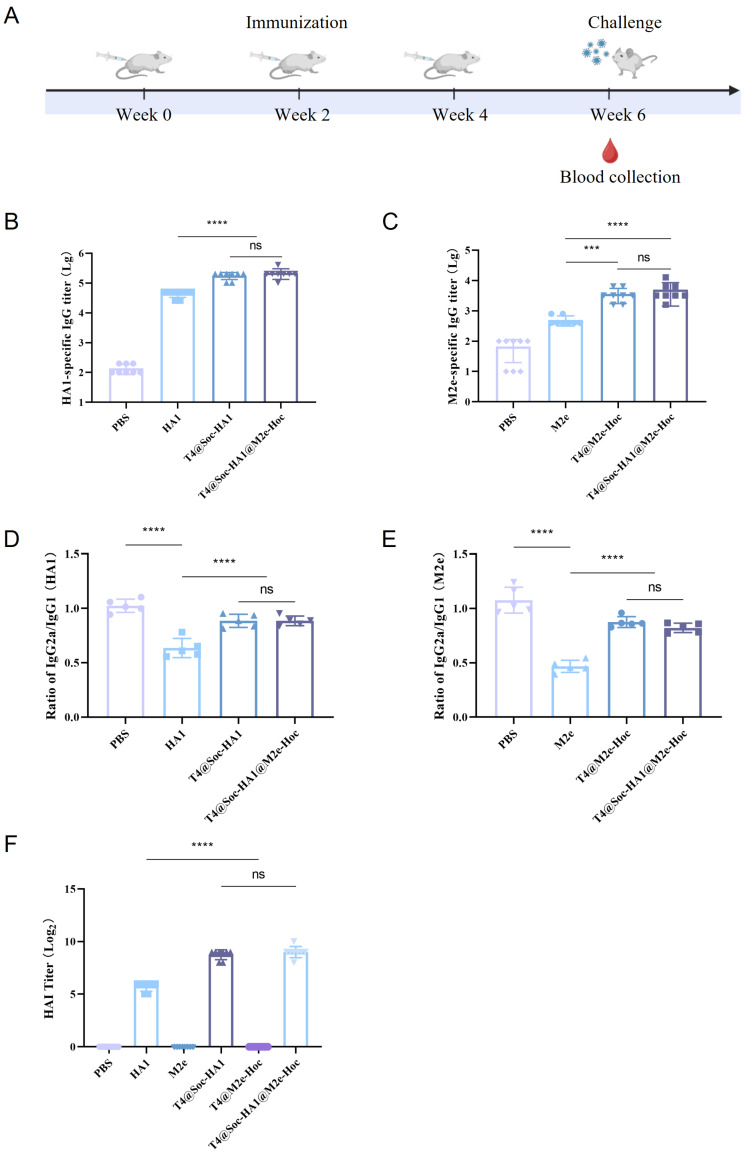
HA1/M2e-specific humoral immune responses. (**A**) Immunization regimen. (**B**) HA1-specific IgG titers measured by ELISA. (**C**) M2e-specific IgG titers measured by ELISA. (**D**) Ratio of HA1-specific IgG2a to IgG1 in serum. (**E**) Ratio of M2e-specific IgG2a to IgG1 in serum. (**F**) HAI antibody titers in serum. Data are represented as mean ± S.D. ***, **** and ns indicate *p* < 0.001, *p* < 0.0001 and not statistically significant, respectively (ANOVA).

To determine the immunogenicity of several nanovaccines based on the T4 phage prepared in this study, 6-week-old BALB/c mice were immunized via intramuscular injection without the use of adjuvants. PBS, soluble antigen HA1, and soluble antigen M2e were used as controls. Serum was collected in the second week following the last immunization to measure specific antibody levels by ELISA (Figure 2A). The results showed that, without adjuvants, all three nanovaccines induced high levels of specific IgG (Figure 2B,C). In contrast, the control group that received PBS showed negative HA1- and M2e-specific IgG antibodies. Although the groups immunized with soluble HA1 and soluble M2e antigens induced specific IgG antibodies, the antibody titers were low (Figure 2B,C).

Typically, IgG1 is closely associated with Th2-type immune responses, while IgG2a is closely linked to Th1-type immune responses [54]. Therefore, to evaluate the type of immune response induced by the vaccines, we further assessed the specific IgG1 and IgG2a ratios in each group to determine the Th1/Th2 immune response. As shown in Figure 2D,E, the specific IgG2a/IgG1 ratios in the two control groups immunized with soluble antigens were much less than 1, indicating that without adjuvants, the humoral immune response induced by soluble antigens was predominantly Th2-biased. Although the specific IgG2a/IgG1 ratios in the sera of mice immunized with the three T4 nanovaccines were still all less than 1, showing a certain tendency towards Th2 immune response, compared with other experimental groups immunized with soluble antigens, their ratios were all closer to 1. This indicates that under certain conditions, the antigens delivered by T4 phages can make the Th1/Th2 immune response generated by the organism more balanced.

Additionally, the Hemagglutination Inhibition (HAI) assay results revealed that the HAI titers of the two nanovaccine groups containing HA1 antigen were significantly higher than those of the group immunized with HA1 alone (Figure 2F), indicating that the antigens displayed on T4 phages elicited a stronger humoral response.

### 3.4. T4 Phage-Based Nanovaccine Elicited a Strong Cellular Immune Response

**Figure 3:**
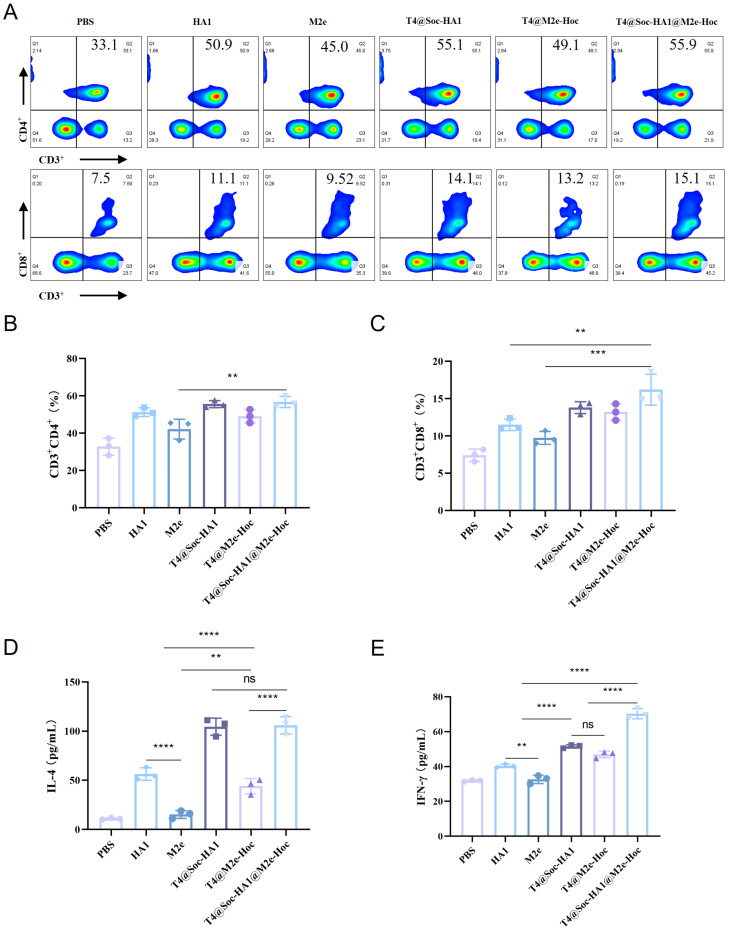
HA1/M2e-specific cellular immune responses. Mice were immunized according to the regimen shown in Figure 2A, and splenocytes were isolated on day 14 after the final immunization. (**A**) Splenocytes isolated from immunized mice were stained with anti-CD3, CD4, and CD8a antibodies and analyzed by flow cytometry. (**B**) Percentage of CD3^+^CD4^+^ T cells within the splenocyte population. (**C**) Percentage of CD3^+^CD8^+^ T cells within the splenocyte population. (**D**) Levels of IL-4 in the culture supernatant of splenocytes. (**E**) Levels of IFN-γ in the culture supernatant of splenocytes. Data are represented as mean ± S.D. **, ***, **** and ns indicate *p* < 0.01, *p* < 0.001, *p* < 0.0001 and not statistically significant, respectively (ANOVA).

To determine whether the three nanovaccines could also induce a strong cellular immune response, mice from each group were randomly selected 14 days after the third immunization. After euthanasia, spleens were harvested to prepare splenocyte suspensions, and different T cell populations within the splenocytes were analyzed using flow cytometry. The results indicated that compared to the control groups, the T4@Soc-HA1@M2e-Hoc nanovaccine induced higher levels of CD3^+^CD4^+^ T cells (Figure 3A,B), and the levels of CD3^+^CD8^+^ T cells were also significantly higher than those in the control groups (Figure 3A,C).Typically, Th2-type immune responses are associated with the secretion of IL-4, while Th1-type immune responses are related to the secretion of IFN-γ [55,56]. Therefore, to better evaluate the immune response, splenocytes from each group were co-cultured with M2e peptide and HA1 protein as stimulants for a certain period of time, and the levels of IL-4 and IFN-γ cytokines in the supernatant were measured. The results showed that the IL-4 cytokine levels in the supernatant of splenocyte cultures from mice immunized with the T4@Soc-HA1@M2e-Hoc nanovaccine were significantly higher than those in the two control groups immunized with soluble antigens (Figure 3D). Similarly, measuring IFN-γ cytokine levels in the same manner revealed that the cytokine levels in the T4@Soc-HA1@M2e-Hoc nanovaccine group were still significantly higher than those in the other control groups (Figure 3E). These cellular immune responses indicate that, in addition to humoral immune responses, the antigens delivered by T4 phages can elicit a stronger T cell immune response.

### 3.5. Nanovaccine Based on T4 Phage Provides Complete Protection Against Lethal Influenza Virus Challenge

**Figure 4:**
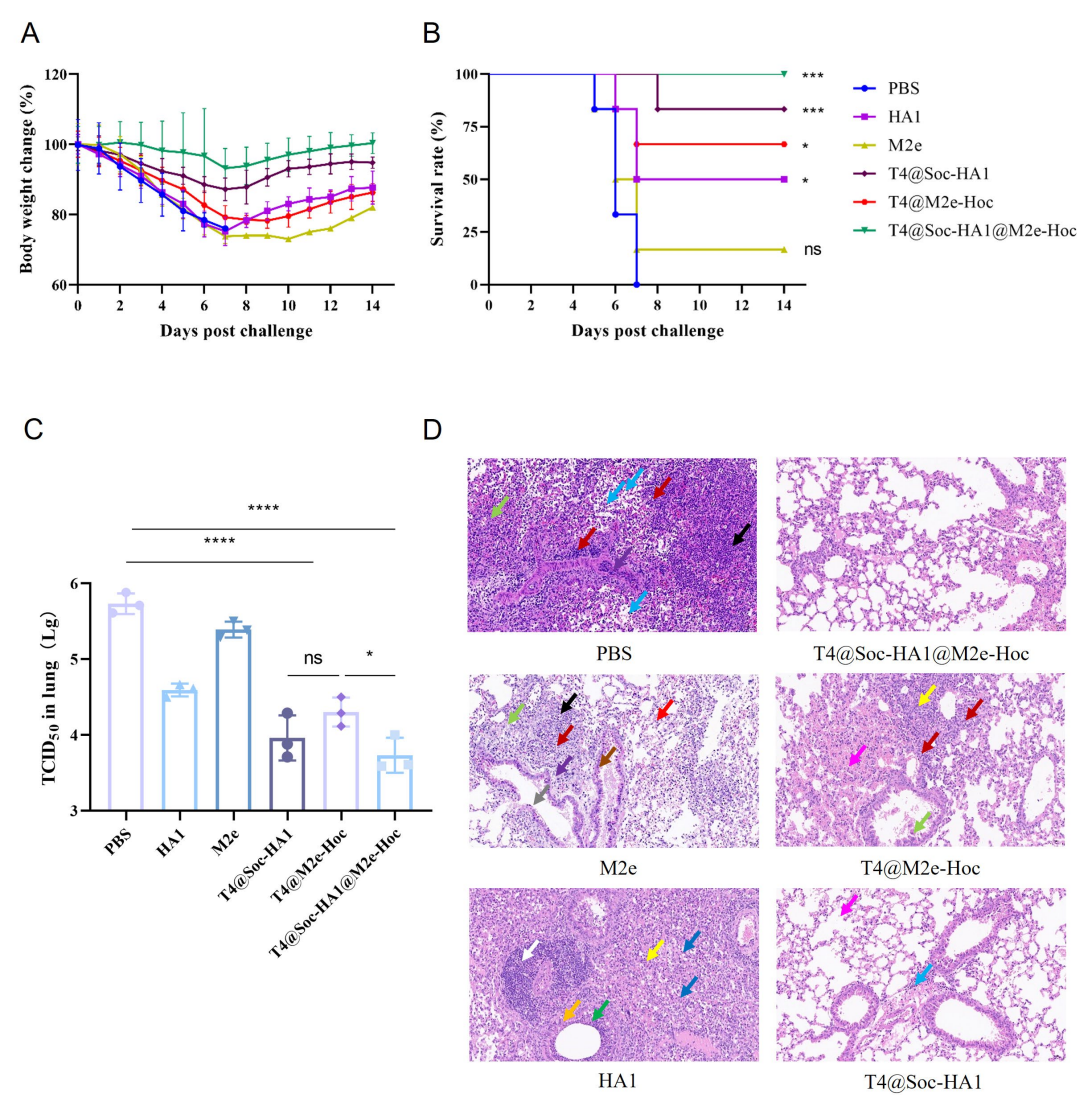
Evaluation of nanovaccine protection efficacy. Mice (*n* = 6) were subjected to a viral challenge two weeks after the final immunization. (**A**) Changes in mice body weight over 14 days post-challenge. (**B**) Survival rates of mice over 14 days post-challenge. (**C**) Lung viral loads 5 days post-challenge. (**D**) Histopathological analysis of the lungs from virus-challenged mice (*n* = 3), which were immunized using the same regimen as described above. Mice were euthanized, and lung sections were prepared as described in the Materials and Methods. Images represent typical results for each group, including multifocal necrosis in lung tissue (black arrows), numerous fibroblasts (light green arrows), a heavy infiltration of lymphocytes and granulocytes (dark red arrows), slight granulocyte infiltration in the alveolar walls (red arrows), abundant lymphocytes, granulocytes, and macrophages within the alveoli (light blue arrows), slight bronchiolar epithelial cell necrosis (gray arrows), necrotic cell debris and eosinophilic material (brown arrows), slight perivascular edema (blue arrows), leukocytes occluding the lumen (purple arrows), necrotic cell debris (yellow arrows), detached epithelial cells (green arrows), a small amount of perivascular lymphocytic infiltration is observed in a ring-like pattern (white arrows), and mild hemorrhage (light purple arrows). The data were analyzed using the Mantel–Cox test. *, ***, **** and ns indicate *p* < 0.05, *p* < 0.001, *p* < 0.0001 and not statistically significant, respectively (ANOVA).

To evaluate the protective effect of the nanovaccine, groups of immunized mice (each consisting of six mice) were challenged with a dose of 5 × LD_50_ of H3N2 influenza virus. The mice were monitored daily for body weight and mortality over a period of 14 days. As shown in Figure 4A, all groups, except for the T4@Soc-HA1@M2e-Hoc group, exhibited significant weight loss between days 1 and 7. In the PBS group, all mice died within 9 days post-challenge. In the M2e group, only one mouse survived after 14 days of challenge, whereas the survival rate in the HA1 group was 50%. In contrast, the three experimental groups immunized with the T4 phage nanovaccine showed an increased survival rate by day 14 post-challenge, with all mice in the T4@Soc-HA1@M2e-Hoc nanovaccine group surviving (Figure 4B). In accordance with related studies, our data were also evaluated and analyzed using GraphPad Prism 8.0 (GraphPad Software) [51].

To further assess the protective effect of each formulation, additional groups of mice immunized with the same regimen and dose were challenged two weeks after the last immunization. On day 5 post-infection, lung tissues were collected for viral load assessment and histopathological analysis. The results indicated that the viral load in the lungs of mice immunized with the T4@Soc-HA1@M2e-Hoc nanovaccine was significantly lower compared to the control groups immunized with soluble antigens (Figure 4C). Furthermore, an histopathological examination of stained lung tissue sections revealed no abnormalities in the lungs of mice immunized with the T4@Soc-HA1@M2e-Hoc nanovaccine (Figure 4D). In contrast, the PBS group and the group immunized with soluble M2e antigen exhibited clear signs of focal necrosis, connective tissue proliferation, the presence of a few fibroblasts, mild hemorrhage, and slight lymphocyte infiltration; alveolar narrowing and variability in alveolar size were also observed. Although other groups showed varying degrees of abnormalities, these were not as pronounced.

## 4. Discussion

The H3N2 influenza virus exhibits high variability and transmissibility, making the development of newer, faster, and more scalable vaccine technologies crucial for rapidly responding to novel influenza outbreaks and pandemics [57]. Previously, studies have shown that a T4 phage virus-like particle vaccine designed by displaying the M2e from influenza A viruses infecting three different species on the T4 phage at a high copy number could resist the attack of the lethal H1N1 virus without using adjuvants, demonstrating a good protective effect [52]. In our current study, we prepared a T4@Soc-HA1@M2e-Hoc nanovaccine via the T4 phage in vitro display technology. This nanovaccine simultaneously displays two antigenic components of the influenza virus, further exploring the potential of the T4 phage as a vaccine platform. Moreover, it can trigger robust humoral and cellular immune responses without the use of adjuvants and provide complete protection for mice against homologous influenza virus attacks.

By co-incubating the obtained fusion proteins Soc-HA1 and M2e-Hoc with Soc^−^Hoc^−^T4 phages, we successfully assembled nanovaccines such as T4@Soc-HA1@M2e-Hoc. In our strategy for constructing nanovaccines, we chose to express and purify both fusion proteins in vitro before assembly. Although this method introduces additional steps for purifying fusion proteins compared to in vivo assembly methods, the “building block” assembly approach we employed significantly enhances flexibility [58]. Therefore, to produce the nanovaccine, we first successfully synthesized two fusion proteins (Figure 1A). Subsequently, we co-incubated the produced fusion proteins with Soc^−^Hoc^−^T4 phages to load the antigens onto the phage (Figure 1B). Compared to the wild-type T4 phage, which displays 810 copies of Soc protein and 155 copies of Hoc protein [59], the display efficiency of the Soc-HA1 and M2e-Hoc fusion proteins is reduced, with the former displaying 179 copies and the latter displaying 68 copies. This result is consistent with findings from other studies utilizing T4 phage display technology [33,60,61]. This reduction can be attributed to several factors. First, the fusion of exogenous proteins may alter the spatial conformation and distribution of Soc and Hoc proteins on the phage surface [62], leading to increased spatial crowding and thereby affecting assembly efficiency. Second, factors related to in vitro expression (such as the characteristics of expression vectors, selection of protein tags, and optimization of purification conditions) can influence the purity, integrity [63], and activity of the fusion proteins, which in turn affects assembly and display copy numbers. Additionally, conditions within the assembly system (such as buffer composition, pH, ionic strength, temperature, and duration) may also impact the interaction between the fusion proteins and the phage coat [64], consequently influencing the display copy numbers.

The antigens loaded onto the T4 phage elicited stronger immune responses even in the absence of adjuvants. As expected, compared to immunization with soluble proteins alone, the T4 phage-based vaccine strategy induced higher levels of specific antibodies against HA1 and M2e antigens post-immunization (Figure 2B,C). This may be attributed to the repetitive and symmetrical binding sites for Soc and Hoc on the T4 phage particle, allowing the HA1 and M2e antigens on the constructed fusion proteins to be presented in a manner similar to the natural viral surface structure, making them more readily recognizable by the immune system [65]. Additionally, the antigens at the high copy numbers displayed on the phage surface form an integral complex with the T4 phage, leveraging the phage’s intrinsic immunostimulatory properties to enhance antigen presentation and immune response in the host [66]. This adjuvant-free strategy for improving antigen immunogenicity is advantageous as it avoids the potential side effects associated with adjuvant use [67,68].

In the ongoing exploration of influenza immunity, two distinct viewpoints have emerged. The first perspective emphasizes the critical importance of a balanced Th1/Th2 response in protecting against influenza infection [52,69], highlighting the cooperative roles of Th1-mediated cell-mediated immunity and Th2-driven humoral immunity in establishing a robust “immune defense network.” When this balance is maintained, the immune system can effectively respond to influenza virus invasion. However, an imbalance may lead to compromised protection; an excessive Th2 skew could result in antibody-mediated immunopathological damage [70], while an extreme Th1 response might hinder effective viral clearance, leading to persistent infection [71]. In contrast, research conducted by Tom Braciale and Suzy Swain since 1994 has pointed out that Th2-polarized effectors are inadequate in defending against influenza, whereas Th1 effectors are pivotal. Despite IFN-γ not being absolutely essential, the cytotoxic activity of Th1 cells and their ability to assist CD8 T cells are crucial protective mechanisms in combating influenza [72,73,74,75,76,77]. We propose that these viewpoints are not mutually exclusive but rather complementary. The balanced Th1/Th2 response offers a macroscopic view of immune defense, while the emphasis on Th1 cells explores specific cellular functional mechanisms. Th1 cells’ protective mechanisms position them as vital in maintaining a balanced Th1/Th2 response, thereby aiding the overall immune system in effectively clearing influenza viruses. After analyzing the IgG2a/IgG1 ratios (Figure 2D,E) as well as the content of IL-4 and IFN-γ (Figure 3D,E), we found that although all the immunized mice exhibited a certain Th2-biased immune response, the Th1/Th2 immune response induced by the antigens delivered by T4 phages was more balanced. As is widely known, BALB/c mice are biased towards humoral immunity, while C57BL/6 mice are biased towards cellular immunity [78], which might be one of the reasons for the mice to show a certain Th2-biased immune response. Whether the above experimental conclusions hold true for C57BL/6 mice will be the content that we need to explore in our next step. Moreover, although the method of directly measuring the IgG2a/IgG1 ratio adopted in this measurement process has certain limitations compared with the method of demonstrating its actual endpoint titer, we have ensured, to some extent, the accuracy and comparability of the data within the same experimental system through strict experimental operation procedures and quality control. In summary, these results provide valuable insights for future studies on the impact of different antigen delivery methods and host backgrounds on the dynamics of immune responses, ultimately contributing to the development of more effective strategies for the prevention and treatment of influenza.

Compared with other types of influenza vaccines, the traditional inactivated influenza vaccine mainly induces Th2-type humoral immune responses, and the antibodies produced are mainly neutralizing antibodies, but it is relatively weak in stimulating cell-mediated immunity [79]. The live attenuated vaccine has certain limitations in its application due to its safety issues and the possible risk of virulence reversion [80]. The high antigen copy number and unique nanoparticle structure of the T4 phage nanovaccine are beneficial to the uptake and processing of antigens by antigen-presenting cells, thereby inducing a comprehensive and coordinated immune response and enhancing the organism’s resistance to influenza viruses. By measuring cytokines, we found that the antigens delivered by phages could stimulate specific cellular immune responses (Figure 3D,E). The production of cytokines such as IL-4 and IFN-γ indicates that the immune system has generated an effective cellular immune response to the antigens displayed by the phage, which is crucial for combating pathogens such as influenza viruses. Cellular immunity plays a key role in clearing infected pathogens and providing long-term immune protection [81].

In the attack protection experiment, our results indicate that the T4@Soc-HA1@M2e-Hoc nanoparticle vaccine containing dual antigens can completely prevent lethal influenza virus attacks (Figure 4B). Additionally, the extent of weight loss and lung tissue pathology following viral invasion was minimal (Figure 4A,D). In contrast, although the other two nanovaccines also showed some protection, they did not provide complete protection. Therefore, a comprehensive analysis of the experimental results from the attack protection experiment indicates that the T4@Soc-HA1@M2e-Hoc nanoparticle vaccine demonstrates greater potential in preventing influenza virus infection. We infer that this potential may become more apparent as the sample size increases. In fact, when the same number of phage particles were used for immunization, the total amounts of the same antigen components in the three nanovaccines were approximately equal. The different experimental results suggest that the quantity of antigens may not be the sole factor for enhancing the protection effect. We hypothesized that the strategy of simultaneously displaying two antigens on a single phage could utilize the synergy of multiple antigenic epitopes, enabling the host to generate a more comprehensive immune response. This might explain the 100% protection efficiency achieved by the T4@Soc-HA1@M2e-Hoc nanovaccine.

Overall, this study successfully constructed a novel nanovaccine that simultaneously displays HA1 and M2e antigen clusters by fusing influenza virus HA1 and M2e with T4 phage Soc and Hoc proteins, respectively, and displaying them on T4 phages. This vaccine exhibits exceptional efficacy in both humoral and cellular immunity. In subsequent challenge protection experiments, it provided complete protection for mice against homologous influenza virus infection, fully demonstrating its powerful protective efficacy in practical applications. This study opens new avenues and directions for the development of novel influenza virus vaccines and holds significant theoretical value and practical significance.

## 5. Conclusions

Our study demonstrates that a dual-displayed T4 phage nanoparticle vaccine elicits robust humoral and cellular immune responses, effectively protecting against H3N2 influenza virus infection. This phage-based nanovaccine platform holds promise as a versatile system for the development of vaccines. However, there is a limitation to this study. T4 phages displaying dual-antigen clusters fully protect mice against H3N2 infection. Whether phage nanovaccines with two broad-spectrum antigens have broad-spectrum anti-influenza virus capability was not part of the experiment. Therefore, we will explore this issue in the next step.

## Data Availability

All data are contained within the article and Supplementary Materials.

## Supplementary Materials

The following supporting information can be downloaded at: www.mdpi.com/article/10.3390/vaccines13010070/s1. Table S1: Amplification and identification primer information; Figure S1: Amplification of target fragment; Figure S2: Protein standard curve (Bradford Method); Figure S3: Determination of phage display efficiency. Supporting information is available from the Wiley Online Library or from the author.
