# Supplementary material for: T4 Phage Displaying Dual Antigen Clusters Against H3N2 Influenza Virus Infection
Source: Vaccines (Basel). 2025 Jan 13;13(1):70. doi: 10.3390/vaccines13010070 (PMC11769387; doi:10.3390/vaccines13010070)
Supplement: Supplementary file 1 [file vaccines-13-00070-s001.zip › vaccines-3330768-supplementary.pdf]

Table S1 Amplification and Identification Primer Information.

Primer	Sequence（5’→3’）
Soc-HA1TY-F	CTACACCAGTAAGTGGTCTCGAGGGAAGTGGCAGCGG TAGTGGTAGTCAAAAAATTCCTGGAAATG
Soc-HA1TY-R	GGTGGTGGTGGTGCTCGATTCTAGTTTGTTTCTCT
HA1TY-F	AGCAAATGGGTCGCGGATCCCAAAAAATTCCTGGA
HA1TY-R	CGGAGCTCGAATTCGGATCTCATCTAGTTTGTTTCTC
Pcold-Hoc-M2e-F	TACCCTCGAGGGATCAATGAGCCTGCTGACC
Pcold-Hoc-M2e-R	GCTTGAATTCGGATCTCATGGATAGGTATA
pET-28aJD-F	ATCTCGATCCCGCGAAATTAAT
pET-28aJD-R	GCCAACTCAGCTTCCTTTCG
pColdJD-F	ACGCCATATCGCCGAAAGG
pColdJD-R	GGCAGGGATCTTAGATTCTG

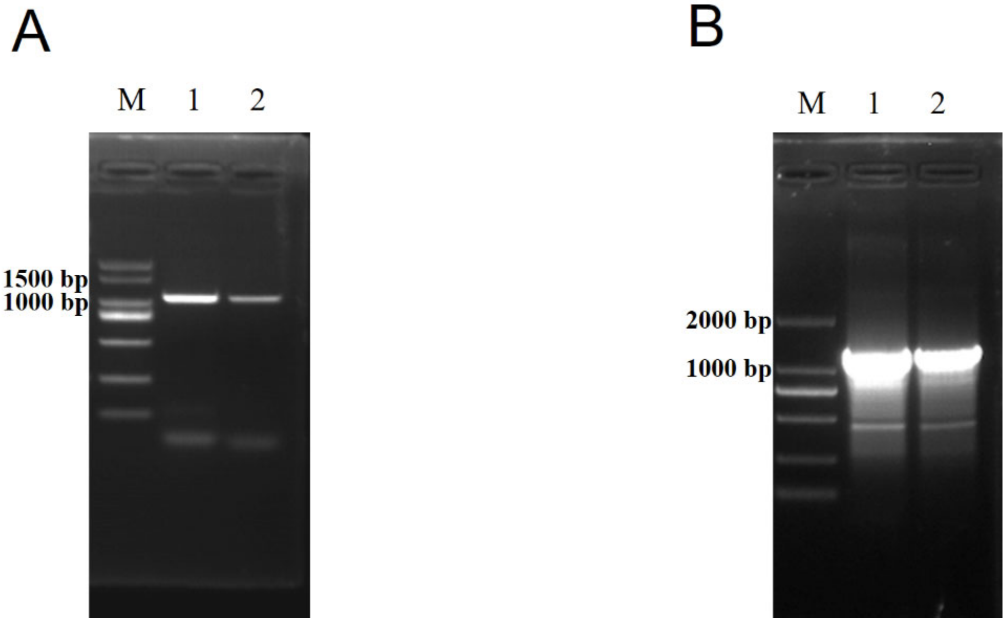

Supplementary Figure 1. Amplification of Target Fragment **(A)** Amplification of the HA1 Gene Fragment (M: DL2000 DNA Maeker,1: Amplification of the Soc-HA1 Gene Fragment,2: Amplification of the HA1 Gene Fragment). **(B)** Amplification of the M2e-Hoc Gene Fragment (M: DL2000 DNA Maeker,1-2: Amplification of the M2e-Hoc Gene Fragment).

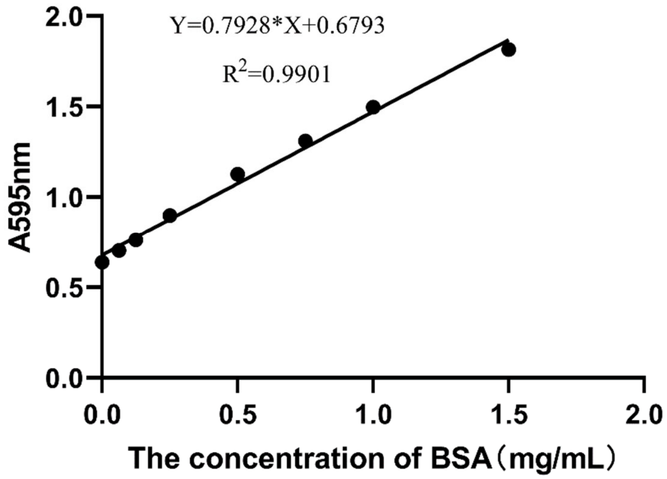

Supplementary Figure 2. Protein Standard Curve (Bradford Method).

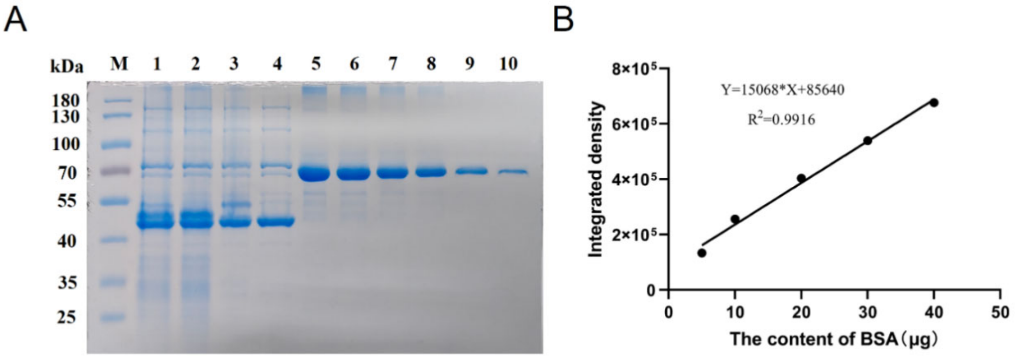

Supplementary Figure 3. Determination of Phage Display Efficiency. **(A)** SDS-PAGE Analysis of Nano-vaccine and Gradient Concentrations of BSA (Lane M: Protein Ladder, Lane 1: T4@Soc-HA1@M2e-Hoc Nano-vaccine, Lane 2: T4@Soc-HA1 Nano-vaccine, Lane 3: T4@M2e-Hoc Nano-vaccine, Lane 4: $Soc^{-}Hoc^{-}$T4 Bacteriophage, Lanes 5-10: Gradient Concentrations of BSA). **(B)** Protein Standard Curve (Densitometry Method).

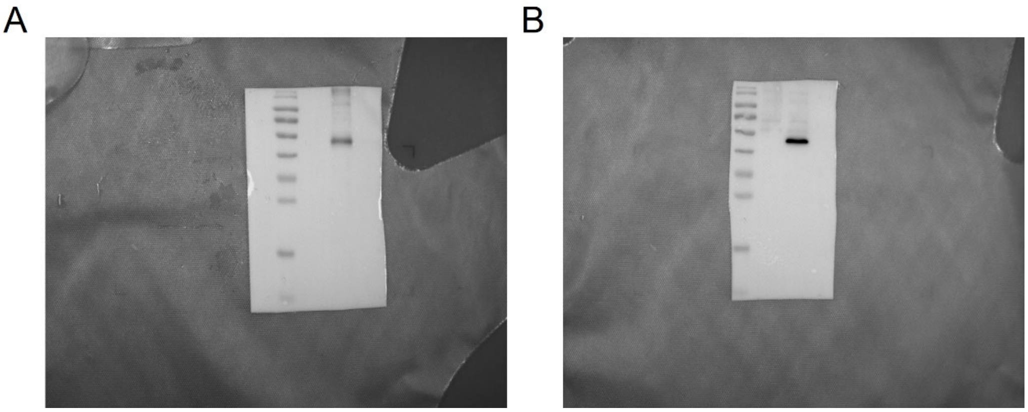

Supplementary Figure 4. The original pictures of Western Blot data in the manuscript:(A) The original picture of Western Blot data in Figure 1C of the manuscript. The densitometry readings of the target band in the picture is 38,619.(B) The original picture of Western Blot data in Figure 1D of the manuscript. The densitometry readings of the target band in the picture is 63,594.

It is worth noting that the Western Blot data is intended to further confirm whether the two fusion proteins can be successfully displayed on Soc$^-$Hoc$^-$T4 Bacteriophage and is only used for qualitative analysis. Since the exposure times of the two pictures are different, it might be rather challenging to obtain more valid data merely by comparing the densitometry readings of the two pictures.
